# Application of machine learning to identify risk factors for outpatient opioid prescriptions following spine surgery

**DOI:** 10.37796/2211-8039.1471

**Published:** 2024-12-01

**Authors:** Alexander Bouterse, Andrew Cabrera, Adam Jameel, David Chung, Olumide Danisa

**Affiliations:** a School of Medicine, Loma Linda University, Loma Linda, CA 92354, USA; b University of California Los Angeles, Los Angeles, CA, 90095, USA; c Department of Orthopedic Surgery, Loma Linda University Medical Center, Loma Linda, CA 92354, USA; d Division of Spine Surgery, Duke University Health, Durham, NC 27710, USA

**Keywords:** Machine learning, Opioid prescriptions, Anterior cervical discectomy and fusion, Laminectomy, Thoracolumbar fusion

## Abstract

**Introduction:**

Spine surgery is a common source of narcotic prescriptions and carries potential for long-term opioid dependence. As prescription opioids play a role in nearly 25 % of all opioid overdose deaths in the United States, mitigating risk for prolonged postoperative opioid utilization is crucial for spine surgeons.

**Purpose:**

The aim of this study was to employ six ML algorithms to identify clinical variables predictive of increased opioid utilization across spinal surgeries, including anterior cervical discectomy and fusion (ACDF), posterior thoracolumbar fusion (PTLF), and lumbar laminectomy.

**Methods:**

A query of the author’s institutional database identified adult patients undergoing ACDF, PTLF, or lumbar laminectomy between 2013 and 2022. Six supervised ML algorithms, including Random Forest, Extreme Gradient Boosting, and LightGBM, were tasked with predicting additional opioid prescriptions at a patient’s first postoperative visit based on set variables. Predictive variables were evaluated for missing data and optimized. Model performance was assessed with common analytical metrics, and variable importance was quantified using permutation feature importance. Statistical analysis utilized Pearson’s Chi-square tests for categorical and independent sample t-tests for numerical differences.

**Results:**

The author’s query identified 3202 patients matching selection criteria, with 841, 1,409, and 952 receiving ACDF, PTLF, and lumbar laminectomy, respectively. The ML algorithms produced an aggregate AUC of 0.743, performing most effectively for lumbar laminectomy. Random Forest and LightGBM classifiers were selected for generation of permutation feature importance (PFI) values. Hospital length of stay was the only highly featured variable carrying statistical significance across all procedures.

**Conclusion:**

Notable risk factors for increased postoperative opioid use were identified, including shorter hospital stays, younger age, and prolonged operative time. These findings can help identify patients at increased risk and guide strategies to mitigate opioid dependence.

## 1. Introduction

Spine surgery is a notable source of narcotic prescriptions and, in some cases, long-term opioid dependence. Specifically, estimates regarding the prevalence of chronic opioid use following spine surgery have ranged from 30 to 60 %, indicating a troubling correlation between postoperative pain management and the severity of the ongoing opioid epidemic [1]. As prescription opioids play a causative role in nearly 25 % of all opioid overdose deaths in the United States, mitigating risk for prolonged postoperative opioid use must be a principal focus for spine surgeons [2].

Prior investigations into the risk factors for chronic postoperative opioid use have identified a number of predictive variables that serve to guide pain management in this cohort of patients. Notably, clinical characteristics including female sex, preoperative opiate use, age, and the presence of psychiatric comorbidities have been found to contribute significantly to increased quantity and duration of opioid prescriptions in the postoperative period [3]. However the degree of invasive dissection and surgical instrumentation varies widely across the diverse catalog of spinal procedures, thus presenting a challenge to providers seeking to quantify the likelihood of this outcome postoperatively. Thus, investigation of risk factors across a number of spinal surgeries may serve to further delineate the characteristics that drive sustained postoperative opioid dependence.

The widespread application of artificial intelligence into medical research invites a deeper exploration of this complex clinical dilemma. Of significance, the use of machine learning (ML) modalities has recently been utilized to effectively isolate and quantify the risk factors associated with an increased duration of post-procedural opioid use [4–7]. Given the insight provided by these studies, further application of ML modalities may be warranted to predict procedural risk factors for increased postoperative opioid prescriptions. As such, the aim of this study was to employ six ML algorithms to identify clinical variables predictive of increased opioid utilization across common spinal surgeries, including anterior cervical discectomy and fusion (ACDF), posterior thoracolumbar fusion (PTLF), and lumbar laminectomy.

## 2. Methods

After receiving a notice of exemption from our institution’s review board (IRB #5240055), Tableau Desktop (Version 2022.4.7, Tableau Software, 2023) was used to query the author’s institutional database in order to identify adult patients undergoing ACDF, PTLF, or lumbar laminectomy between 2013 and 2022. These procedures were selected based upon their relatively high prevalence within our dataset as well as the differences in procedural complexity, instrumentation, and invasive dissection inherent to each. Within this population, International Classification of Disease (ICD) diagnostic codes were utilized to identify comorbid medical conditions and substance use disorders such as a history of opiate abuse. Our primary outcome, postoperative outpatient opioid prescriptions, was defined as any documented opioid prescription occurring at an outpatient visit within two weeks of the patient’s index surgery.

Six supervised ML classification algorithms were constructed in the python programming language using the SciKit-Learn library and the XGboost, LightGBM, and CatBoost packages [8–12]. These algorithms included Random Forest Classifier (RF), Extreme Gradient Boosting Classifier (XGB), Light Gradient-Boosting Machine (LightGBM), Categorical Boosting (CatBoost), Multilayer Perceptron Classifier (MLP), and a logistic regression model (LogReg). These algorithms were tasked with predicting additional opioid prescriptions at a patients first postoperative visit based on a given set of patient variables, including demographics, comorbidities, and preoperative lab values (Tables 1–3).

Prior to ML analysis, the predictive variables were systematically evaluated for missing data. To address missing data within each variable, the missForest package in the R statistical programming language was applied for imputation of the missing data [13,14]. Following data imputation, predictive variables underwent preprocessing using SciKit-Learn’s Robust and Min–Max scalers for continuous variables (eg, age, BMI, lab values) and SciKit-Learn’s OneHotEncoder for categorical variables (eg, race, insurance status) [9]. Our target variable, additional opioid prescriptions at a patient’s first postoperative visit, was encoded as a binary variable. An 80:20 train test split using Scikit-Learn’s train_test_split method was performed in which 80 % of our population data was randomly divided into our training dataset and the remaining 20 % set aside for the final testing of ML model performance on unseen data [9,15]. Training of each algorithm involved utilization of the Tree-Structured Parzen Estimator algorithm for Bayesian optimization from the Opunta library along with a stratified 5-fold cross validation to determine optimal hyperparameters on the training data and ensure model generalizability [16]. Once the appropriate hyperparameters were determined, the final models were subsequently evaluated using the set aside testing data from the train test split to determine the model’s performance.

The performance of the five ML models were then evaluated by a series of commonly used metrics, including classification accuracy, sensitivity, specificity, Positive Predictive Value (PPV), Negative Predictive Value (NPV), F1 score, Area Under Receiver Operating Characteristics Curve (AUROC), and Area Under Precision-Recall Curve (AUPRC) [17–19]. The Matplotlib library in python was used for Graphical visualization of the ROCs and PRCs produced by the six models [20]. Importance of each variable was quantified using permutation feature importance (PFI) through utilization of SciKit-Learn’s permutation importance feature method [14,21]. Generation of PFI values is performed by measurement of model performance when a single variable is removed, disrupting the relationship between the variable and the predicted outcome. Thus, variables attributed with greater PFI values are deemed more important as predictive performance of that model decreases when that variable is removed [21,22]. As our study utilized multiple ML algorithms, the PFIs from the top performing model from each procedure were chosen for further statistical analysis.

Statistical analysis was performed using SPSS version 28 (IBM Corporation, 2021, Armonk, NY, USA) with statistical significance defined as of p < 0.05. Differences between categorical variables were assessed using the Person’s Chi-square test, while numerical differences were assessed using independent sample *t*-test. Categorical variables are presented as frequencies in percentages and continuous variables are presented as means and standard deviations (SD).

## 3. Results

Query of the author’s institutional database identified 3.202 patients matching inclusion criteria that underwent spinal surgery between 2013 and 2022. Among this cohort 841, 1,409, and 952 patients received ACDF, PTLF, and lumbar laminectomy, respectively. In total, 59.0 % of patients within this population were prescribed additional postoperative opioids at their first outpatient appointment, with the highest rate of prescriptions observed among patients undergoing ACDF (71.1 %), followed by PTLF (55.8 %), and lumbar laminectomy (53.2 %). An average of 997.41 MME’s were prescribed in the outpatient setting, with patients undergoing PTLF requiring a comparatively higher quantity of opioids to manage postoperative pain. A full description of the clinical characteristics and outcomes observed by each cohort of patients is provided in Tables 1–3.

**Fig. 1:**
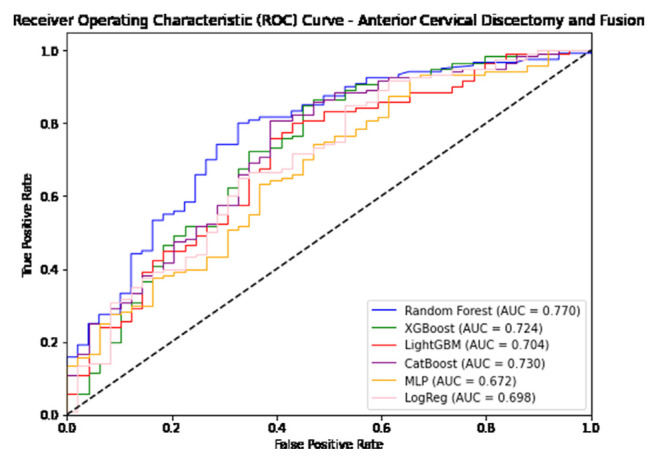
Graphical representation of each algorithms AUROC in prediction of additional opioid prescriptions at a patient’s first postoperative visit following anterior cervical discectomy and fusion.

**Fig. 2:**
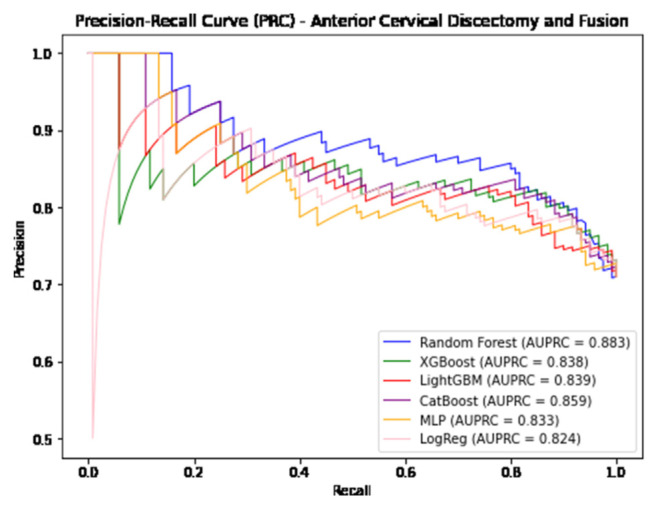
Graphical representation of each algorithms AUPRC in prediction of additional opioid prescriptions at a patient’s first postoperative visit following anterior cervical discectomy and fusion.

**Fig. 3:**
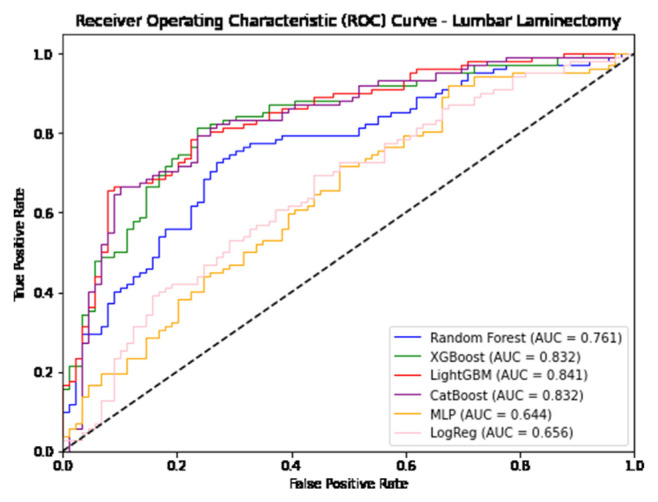
Graphical representation of each algorithms AUROC in prediction of additional opioid prescriptions at a patient’s first postoperative visit following lumbar laminectomy.

**Fig. 4:**
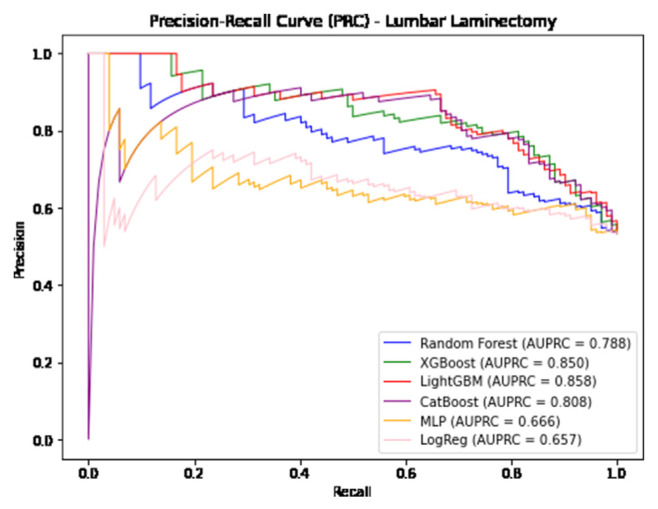
Graphical representation of each algorithms AUPRC in prediction of additional opioid prescriptions at a patient’s first postoperative visit following lumbar laminectomy.

**Fig. 5:**
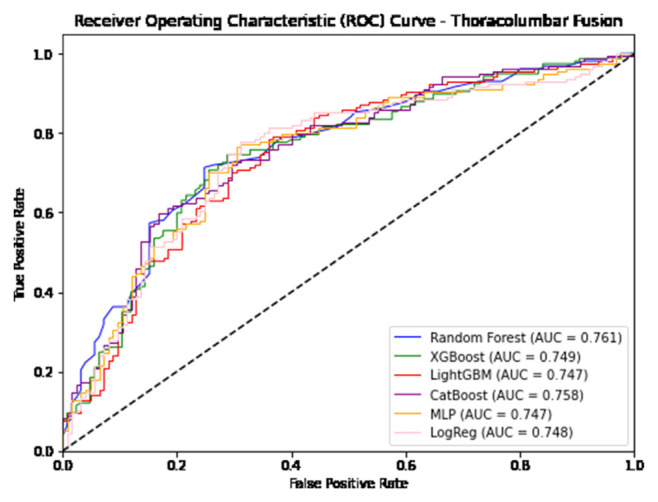
Graphical representation of each algorithms AUROC in prediction of additional opioid prescriptions at a patient’s first postoperative visit following Thoracolumbar Fusion.

**Fig. 6:**
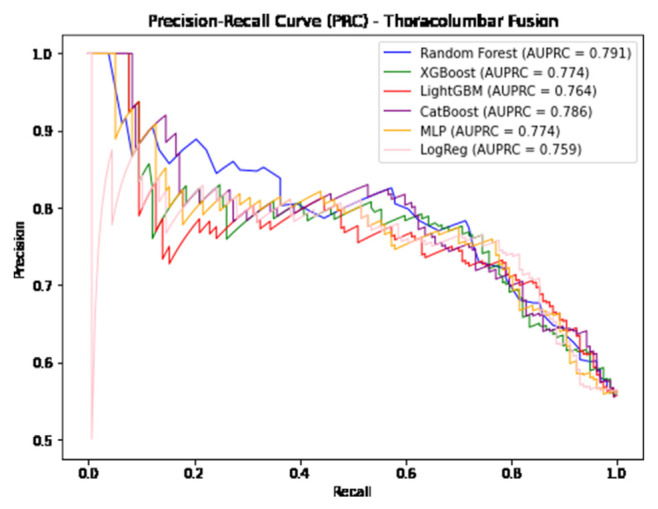
Graphical representation of each algorithms AUPRC in prediction of additional opioid prescriptions at a patient’s first postoperative visit following Thoracolumbar Fusion.

In predicting postoperative opioid prescriptions, the ML classification algorithms produced an aggregate AUC value of 0.743, performing most effectively when applied to the cohort of patients undergoing lumbar laminectomy. For the generation of variable PFI values, Random Forest and Light GBM algorithms were selected based on their comparatively high level of performance in predicting postoperative opioid prescriptions. Further characterization of the performance of each algorithm employed in our analysis is provided in Tables 4–6 and Figs. 1–6.

Application of the RF classifier to patients undergoing ACDF identified hospital length of stay (PFI = 0.2174; p < 0.001), preoperative white blood cell count (PFI = 0.0151; p = 0.006), age (PFI = 0.0118; p = 0.022), operative time (PFI = 0.0084; p = 0.010) preoperative hemoglobin (PFI = 0.0057; p = 0.009), smoking history (PFI = 0.0045; p = 0.029), and preoperative creatinine (PFI = 0.0022; p < 0.001) as statistically significant predictors of additional opioid prescriptions postoperatively. Additional predictive variables identified within this cohort are described in Table 7.

Through utilization of the Light GBM classifier to predict opioid prescriptions in patients receiving lumbar laminectomy, length of stay (PFI = 0.1217; p < 0.001), preoperative platelet levels (PFI = 0.0343; p = 0.043), chronic kidney disease (PFI = 0.0058; p < 0.001), and private insurance coverage (PFI = 0.0056; p = 0.019) were identified as statistically significant predictive variables. Additional predictive variables identified in this group are listed in Table 8.

Finally, in utilizing the RF classifier to predict postoperative opioid prescriptions following PTLF identified the following variables as carrying both statistical significance and notable predictive importance: length of stay (PFI = 0.1178; p < 0.001), age (PFI = 0.0300; p < 0.001), operative time (PFI = 0.0128; p < 0.001), preoperative glucose (PFI = 0.0069; p = 0.048), preoperative creatinine (PFI = 0.0044; p = 0.026), APR Risk of Mortality Classification ≥3 (PFI = 0.0012; p < 0.001), and marital status (PFI = 0.0005; p = 0.019). Further description of the predictive variables identified in this group is provided in Table 9.

## 4. Discussion

As the prevalence of opioid use disorders continues to rise throughout the United States, orthopedic providers must become increasingly aware of clinical risk factors that drive prolonged use of prescription opioids among surgical populations [23]. This study applies ML-based predictive analysis in order to isolate procedure-specific variables associated with additional opioid prescriptions at initial postoperative visits for patients undergoing spine surgery. In identifying and quantifying the contributions of relevant risk factors, providers may better anticipate the likelihood of prolonged opioid requirements and implement strategies to mitigate these factors among high-risk patient populations.

This study serves to highlight several patterns of opioid prescriptions among our selected surgical populations. Most notably, across all procedural groups, a shorter duration of hospitalization was associated with a statistically significant increase in outpatient opioid prescriptions. While the constellation of clinical factors contributing to this phenomenon is not clearly defined, it is possible that this finding reflects a pattern of deferring postoperative pain management regimens to the outpatient setting following routine spine procedures. For example, as the safety of performing minor spine surgeries such as ACDF within an ambulatory setting has become increasingly recognized, many providers have transitioned to same-day discharges, thus eliminating the previously requisite period of inpatient postoperative pain assessment and shifting this requirement to the first postoperative visit [24,25]. While this approach has proven beneficial from both a clinical and financial perspective, this study illustrates that, in some scenarios, it may come at the cost of increasing outpatient opioid requirements. While reports on this phenomenon are inconsistent, it is certainly not without risks as outpatient opioid prescriptions have been associated with increased healthcare expenditures, larger prescription quantities, and a heightened risk for future substance use disorders and opioid overdose [26–28]. Furthermore, as persistent postoperative opioid use is a common and often unrecognized issue, deferring this requirement to the outpatient setting may exacerbate this phenomenon amongst same-day surgery candidates [28–30]. As such, it is important for providers to recognize the trend toward shorter lengths of stay as a potential nidus for increased opioid prescriptions as sameday surgery continues to become popular for low-risk spine surgeries.

Similarly, among several surgical groups examined in our analysis, younger patients and those with prolonged operative times were more frequently the recipients of additional opioids at their first postoperative visit. Such factors have been well-documented within the current literature as notable risk factors, with authors positing that an increased prevalence of substance use disorders among younger patients may drive increasing opioid requirements, while the increased procedural complexity and extent of anatomic dissection associated with longer procedures may worsen postoperative pain [30–32]. This study serves to reinforce these variables as relevant risk factors following spine surgery and emphasizes the importance of recognizing the risk of increased or prolonged opioid use among patients with these characteristics.

With regard to procedure-specific considerations, marriage and private insurance coverage were among those identified as risk factors for PTLF and laminectomy, respectively. Though each carried relatively low feature importance within our study and ultimately may lack clinical significance, it is worth noting that these variables have been previously documented as risk factors for increased or prolonged opioid prescriptions. Specifically, in a review of opioid prescribing patterns following traumatic injuries among military members, Chaudhary et al. found marriage to carry a consistent association with an increased likelihood of opioid prescriptions upon hospital discharge [32]. Similarly, upon review of prescription patterns amongst privately insured and Medicare-eligible patients, Mikosz et al. noted both a longer duration and increased quantity of opioid prescriptions in privately insured patients following lumbar decompression and spinal fusion procedures [33]. Though the identification of these same factors within our study may serve to add credence to their clinical relevance, the consistency of these findings across the literature is not well-supported and may simply be a byproduct of the specific populations examined by these studies. As such, further research is required to better determine the clinical importance of these variables and what causal relationship they may share with the patterns of opioid prescription observed in our study.

In line with other studies that have examined the topic of postoperative opioid prescriptions following spine surgery, our study effectively applied ML algorithms in order to identify risk factors for outpatient prescriptions following a subset of minor procedures. In an examination of prescription patterns following ACDF, Karhade et al. applied a set of ML classifiers to predict sustained postoperative opioid use, achieving AUC values ranging from 0.63 to 0.80 [4]. Similarly, Chen et al. sought to apply this same methodology to a cohort of patients undergoing surgery for lumbar disc herniations in Taiwan and subsequently yielded AUC values between 0.66 and 0.76 [7]. Our study generated comparable predictive efficacy, producing an average AUC value of 0.743 across all utilized algorithms. As such, our examination of this topic provides an equivalent degree of algorithmic performance and serves to elucidate a broadened profile of risk factors for postoperative opioid use.

The results of our analysis must be viewed in light of several key limitations. In particular, through our use of a deidentified institutional database, our results are limited in their scope, comprising only the outcomes of a single patient population. Furthermore, the use of this data source limits our access to variables that may be relevant to a patient’s perioperative course, as the database may be unable to capture these items or may feature a high volume of missing data points. For example, the number of vertebral segments spanned by each procedure, although potentially influential to postoperative opioid requirements, was not readily available within our selected database. Similarly, our insight into outpatient opioid prescriptions was limited to those occurring within two weeks of a patient’s index procedure as this outcome was inconsistently supplied by our data source outside this time range. Additionally, as with all large clinical datasets, the data utilized for our analysis may be confounded by errors that occur during the process of encoding clinical encounters. Finally, it should be qualified that, although a widely utilized method within ML analyses, PFI provides an indirect measure of the relative importance of the perioperative variables contained within our dataset [22,34]. Utilization of this method may predispose our analysis to bias as the permutation of highly correlated variables may inadvertently alter the predictive power of correlated features, thus leading to inaccurate assignment of variable importance [34,35]. As such, though our analysis may serve to highlight proposed risk factors, its findings should be used to supplement clinical expertise rather than to guide perioperative decision-making.

## 5. Conclusions

This study presents the employment of six ML algorithms to predict postoperative opioid prescriptions within the outpatient setting following three common spine surgeries, ACDF, PTLF, and lumbar laminectomy. Through the use of this methodology, notable risk factors for an increase in postoperative opioid requirements were identified, including a shorter duration of hospitalization, younger age, and prolonged operative time. These findings may serve to identify patients at increased risk for persistent opioid use, and, in turn, allow providers to implement strategies to mitigate this risk within the perioperative period.

## Figures and Tables

**Table 1:** Population characteristics – Anterior Cervical Discectomy and Fusion (n = 841).

Characteristic	Mean (+SD) or Percentage (n)
**Age (years)**	55.69 ± 13.61
**Sex**	
*Male*	51.5 % (443)
*Female*	48.5 % (408)
**Race**	
*Asian*	2.0 % (17)
*African American or Black*	8.7 % (73)
*Hispanic*	18.7 % (157)
*Native American or Alaska Native*	0.8 % (7)
*Caucasian or White*	64.4 % (542)
*Unknown or Not Specified*	5.4 % (45)
**Marital status**	
*Married*	54.3 % (448)
*Single*	46.7 % (393)
**Insurance status**	
*Government*	57.6 % (484)
*Private*	41.6 % (350)
*Uninsured*	0.8 % (7)
**Body Mass Index (kg/m** ^**2**^ **)**	30.18 ± 8.24
**Comorbidities**	
*History of smoking*	53.6 % (451)
*CHF*	4.9 % (41)
*COPD*	6.9 % (58)
*CKD*	4.4 % (37)
*Diabetes*	15.0 % (126)
*Chronic Steroid Use*	2.5 % (21)
*Opioid Use History*	5.1 % (43)
**Preoperative Lab Values**	
*Hemoglobin*	12.95 ± 1.67
*White Blood Cell Count*	11.05 ± 3.56
*Platelet Count*	243.67 ± 63.19
*Sodium*	128.47 ± 2.58
*Potassium*	4.08 ± 0.40
*Creatinine*	0.87 ± 0.57
*Glucose*	129.49 ± 43.24
**All Patient Refined Diagnosis-Related Group Risk of Morality**	
*Minor*	74.1 % (623)
*Moderate*	14.1 % (119)
*Major*	5.9 % (50)
*Extreme*	5.8 % (49)
**Operative Time (minutes)**	170.46 ± 94.60
**Length of stay (days)**	4.90 ± 8.03
**Milligram Equivalents of Morphine**	925.3
**Outpatient Opioid Prescription**	
*No*	28.9 % (243)
*Yes*	71.1 % (598)

**Table 2:** Population characteristics – Lumbar Laminectomy (n = 952).

Characteristic	Mean (±SD) or Percentage (n)
**Age (years)**	57.91 ± 16.11
**Sex**	
*Male*	54.4 % (518)
*Female*	45.6 % (434)
**Race**	
*Asian*	3.2 % (30)
*African American or Black*	10.1 % (96)
*Hispanic*	22.7 % (216)
*Native American or Alaska Native*	0.3 % (3)
*Caucasian or White*	59.6 % (567)
*Unknown or Not Specified*	4.1 % (39)
**Marital status**	
*Married*	55.5 % (528)
*Single*	44.5 % (424)
**Insurance status**	
*Government*	64.3 % (612)
*Private*	34.3 % (327)
*Uninsured*	1.4 % (13)
**Body Mass Index (kg/m** ^**2**^ **)**	30.88 ± 8.48
**Comorbidities**	
*History of smoking*	48.1 % (458)
*CHF*	5.4 % (51)
*COPD*	6.2 % (59)
*CKD*	10.3 % (98)
*Diabetes*	16.2 % (154)
*Chronic Steroid Use*	3.6 % (34)
*Opioid Use History*	5.3 % (50)
**Preoperative Lab Values**	
*Hemoglobin*	12.50 ± 1.80
*White Blood Cell Count*	10.62 ± 3.54
*Platelet Count*	242.85 ± 76.37
*Sodium*	137.86 ± 2.90
*Potassium*	4.08 ± 0.43
*Creatinine*	0.90 ± 0.74
*Glucose*	128.76 ± 43.87
**All Patient Refined Diagnosis-Related Group Risk of Morality**	
*Minor*	73.3 % (698)
*Moderate*	16.4 % (156)
*Major*	6.6 % (63)
*Extreme*	3.7 % (35)
**Operative Time (minutes)**	154.9 ± 87.0
**Length of stay (days)**	5.6 ± 7.1
**Milligram Equivalents of Morphine**	688.44
**Outpatient Opioid Prescription**	
*No*	46.8 % (446)
*Yes*	53.2 % (506)

**Table 3:** Population characteristics – Posterior Thoracolumbar Fusion (n = 1409).

Characteristic	Mean (±SD) or Percentage (n)
**Age (years)**	57.02 ± 16.70
**Sex**	
*Male*	49.0 % (690)
*Female*	51.0 % (719)
**Race**	
*Asian*	3.0 % (42)
*African American or Black*	8.6 % (121)
*Hispanic*	26.4 % (372)
*Native American or Alaska Native*	0.5 % (7)
*Caucasian or White*	58.1 % (818)
*Unknown or Not Specified*	3.1 % (44)
**Marital status**	
*Married*	50.7 % (714)
*Single*	49.3 % (695)
**Insurance status**	
*Government*	67.8 % (955)
*Private*	30.8 % (434)
*Uninsured*	1.4 % (20)
**Body Mass Index (kg/m** ^**2**^ **)**	30.18 ± 8.002
**Comorbidities**	
*History of smoking*	51.3 % (723)
*CHF*	7.9 % (112)
*COPD*	8.2 % (115)
*CKD*	6.1 % (86)
*Diabetes*	15.5 % (218)
*Chronic Steroid Use*	6.3 % (89)
*Opioid Use History*	3.0 % (42)
**Preoperative Lab Values**	
*Hemoglobin*	11.66 ± 2.11
*White Blood Cell Count*	11.40 ± 5.60
*Platelet Count*	228.33 ± 77.76
*Sodium*	138.30 ± 2.31
*Potassium*	3.98 ± 0.47
*Creatinine*	0.86 ± 0.66
*Glucose*	125.31 ± 42.06
**All Patient Refined Diagnosis-Related Group Risk of Morality**	
*Minor*	62.5 % (881)
*Moderate*	18.7 % (263)
*Major*	11.6 % (164)
*Extreme*	7.2 % (101)
**Operative Time (minutes)**	255.73 ± 123.89
**Length of stay (days)**	7.72 ± 7.64
**Milligram Equivalents of Morphine**	1249.13
**Outpatient Opioid Prescription**	
*No*	44.2 % (623)
*Yes*	55.8 % (786)

**Table 4:** Algorithm predictive performance – anterior cervical discectomy and fusion.

	Accuracy	Sensitivity	Specificity	PPV	NPV	F1	AUROC	AUPRC
Random Forest	76.33 %	0.8083	0.6531	0.8509	0.5818	0.8291	0.7700	0.8825
XGBoost	76.92 %	0.9083	0.4286	0.7956	0.6563	0.8482	0.7245	0.8375
LightGBM	72.19 %	0.875	0.3469	0.7664	0.5313	0.8171	0.7037	0.8391
Catboost	75.15 %	0.925	0.3265	0.7708	0.6400	0.8409	0.7304	0.8591
Multilayer Perceptron	73.37 %	0.8917	0.3469	0.7698	0.5667	0.8263	0.6721	0.8331
Logistic Regression	75.74 %	0.9167	0.3673	0.7801	0.6429	0.8429	0.6978	0.8241
*Average*	***74.95 %***	***0.8875***	***0.4116***	***0.7889***	***0.6032***	***0.8341***	***0.7164***	***0.8459***

**Table 5:** Algorithm predictive performance – lumbar laminectomy.

	Accuracy	Sensitivity	Specificity	PPV	NPV	F1	AUROC	AUPRC
Random Forest	72.25 %	0.7059	0.7416	0.7579	0.6875	0.7310	0.7612	0.7880
XGBoost	78.01 %	0.8235	0.7303	0.7778	0.7831	0.7998	0.8322	0.8501
LightGBM	77.49 %	0.7843	0.7640	0.7921	0.7556	0.7882	0.8410	0.8575
Catboost	77.49 %	0.7843	0.7640	0.7921	0.7556	0.7882	0.8321	0.8084
Multilayer Perceptron	61.26 %	0.7255	0.4831	0.6167	0.6056	0.6667	0.6444	0.6658
Logistic Regression	61.26 %	0.7745	0.4270	0.6810	0.6230	0.6810	0.6559	0.6574
*Average*	***71.29 %***	***0.7663***	***0.6517***	***0.7363***	***0.7017***	***0.7425***	***0.7611***	***0.7712***

**Table 6:** Algorithm predictive performance – posterior thoracolumbar fusion.

	Accuracy	Sensitivity	Specificity	PPV	NPV	F1	AUROC	AUPRC
Random Forest	70.92 %	0.7325	0.6800	0.7419	0.6693	0.7372	0.7615	0.7912
XGBoost	69.15 %	0.8153	0.5360	0.6882	0.6979	0.7464	0.7492	0.7743
LightGBM	70.57 %	0.7452	0.6560	0.7313	0.6721	0.7382	0.7467	0.7643
Catboost	70.57 %	0.7707	0.6240	0.7202	0.6842	0.7446	0.7576	0.786
Multilayer Perceptron	71.28 %	0.7898	0.6160	0.7209	0.7000	0.7538	0.7472	0.7742
Logistic Regression	72.34 %	0.8344	0.5840	0.7158	0.7374	0.7706	0.7481	0.7589
*Average*	***70.81 %***	***0.7813***	***0.6160***	***0.7197***	***0.6935***	***0.7485***	***0.7517***	***0.7748***

**Table 7:** Random forest permutation feature importance – Anterior Cervical Discectomy and Fusion.

Features	Permutation Feature Importance	Mean ± SD or % Sample (n) – No Postoperative Prescription	Mean ± SD or % Sample (n) – Postoperative Prescription	*p*
*Length of stay (days)*	0.2174	9.51 ± 12.61	3.02 ± 3.76	<**0.001**
*Preoperative Glucose (mg/dL)*	0.0163	132.24 ± 50.26	128.37 ± 40.04	0.239
*Preoperative White Blood Cell Count (x10* ^*3*^ */μl)*	0.0151	10.52 ± 3.43	11.27 ± 3.60	**0.006**
*Body Mass Index (kg/m* ^*2*^ *)*	0.0129	30.33 ± 8.40	30.26 ± 8.18	0.914
*Age (years)*	0.0118	57.38 ± 15.29	55.01 ± 12.81	**0.022**
*White Race*	0.0085	63.79 % (155)	64.72 % (387)	0.799
*Optime*	0.0084	182.70 ± 114.48	165.08 ± 84.72	**0.01**
*Preoperative Hemoglobin (g/dL)*	0.0057	12.71 ± 1.87	13.04 ± 1.58	**0.009**
*History of smoking*	0.0045	47.74 % (116)	56.02 % (335)	**0.029**
*Preoperative Creatinine*	0.0022	0.99 ± 0.87	0.82 ± 0.38	<**0.001**

Bold values denotes statistical significance.

**Table 8:** LightGBM permutation feature importance – Lumbar Laminectomy.

Features	Permutation Feature Importance	Mean ± SD or % Sample (n) – No Postoperative Prescription	Mean ± SD or % Sample (n) – Postoperative Prescription	*p*
*Length of stay (days)*	0.1217	6.70 ± 8.29	4.67 ± 8.75	<**0.001**
*Preoperative White Blood Cell Count (x10* ^*3*^ */μl)*	0.0485	10.59 ± 3.40	10.65 ± 3.66	0.791
*Preoperative Creatinine (mg/dL)*	0.0479	0.93 ± 0.91	0.88 ± 0.54	0.267
*Preoperative Glucose (mg/dL)*	0.0459	130.68 ± 45.84	127.06 ± 42.02	0.203
*Body Mass Index (kg/m* ^*2*^ *)*	0.0445	30.99 ± 8.16	30.77 ± 8.75	0.686
*Operative Time (minutes)*	0.0357	153.82 ± 92.01	155.87 ± 82.38	0.716
*Preoperative Platelet Count (x10* ^*3*^ */μl)*	0.0343	248.19 ± 77.84	238.15 ± 74.80	**0.043**
*Age (years)*	0.0289	58.99 ± 16.14	56.96 ± 16.04	0.053
*History of Chronic Kidney Disease*	0.0058	15.25 % (68)	5.93 % (30)	<**0.001**
*Private Insurance Coverage*	0.0056	30.50 % (136)	37.75 % (191)	**0.019**

Bold values denotes statistical significance.

**Table 9:** Random forest permutation feature importance – Posterior Thoracolumbar Fusion.

Features	Permutation Feature Importance	Mean ± SD or % Sample (n) – No Postoperative Prescription	Mean ± SD or % Sample (n) – Postoperative Prescription	*p*
*Length of stay (days)*	0.1178	9.79 + 9.50	6.08 + 5.20	<**0.001**
*Age (years)*	0.0300	58.90 + 15.99	55.53 + 17.10	<**0.00**1
*Preoperative Platelet Count (x10* ^*3*^ */μl)*	0.0054	229.01 + 80.34	227.80 + 75.70	0.774
*Operative Time (minutes)*	0.0128	273.87 + 138.56	241.34 + 109.04	<**0.001**
*Body Mass Index (kg/m* ^*2*^ *)*	0.0063	29.97 + 8.08	30.34 + 7.97	0.388
*Preoperative Glucose (mg/dL)*	0.0069	127.80 + 43.62	123.33 + 40.71	**0.048**
*Preoperative Creatinine (mg/dL)*	0.0044	0.90 + 0.80	0.82 + 0.52	**0.026**
*Preoperative White Blood Cell Count (x10* ^*3*^ */μl)*	0.0015	11.65 + 2.14	11.67 + 2.09	0.844
*APR Risk of Mortality* – *Major*	0.0012	19.10 % (119)	5.73 % (45)	<**0.001**
*Marital status* – *Married*	0.0005	47.19 % (294)	53.44 % (420)	**0.019**

Bold values denotes statistical significance.
